# How to monitor and discriminate the causes of lower limb swelling during home-based rehabilitation after total knee arthroplasty? A delphi study

**DOI:** 10.1186/s42836-024-00285-9

**Published:** 2025-01-07

**Authors:** Lin Yang, Hui-Wu Li, Zan-Jing Zhai, Cai-Feng Wang, Bei-Ying Wu, Jia Zhou, Wei-Wei Bian, Hong Ruan

**Affiliations:** 1 Department of Nursing, Shanghai Ninth People’s Hospital, Shanghai Jiao Tong University School of Medicine, Shanghai, 200023 China; 2 School of Nursing, Shanghai Jiao Tong University, Shanghai, 200011 China; 3 Department of Orthopaedic, Shanghai Ninth People’s Hospital, Shanghai Jiao Tong University School of Medicine, Shanghai, 200023 China

**Keywords:** Total knee arthroplasty, Swelling, Symptom monitor, Home-based rehabilitation, Delphi method

## Abstract

**Purpose:**

Swelling in the lower limbs after total knee arthroplasty (TKA) affects surgical outcomes. Prolonged swelling requires monitoring and remote management during home-based rehabilitation. Causes of swelling vary but, so far, no indicators are available to monitor and identify causes of lower limb swelling, making it difficult to implement targeted interventions. This study aimed to find the indicators to monitor and identify the causes of lower limb swelling during home-based rehabilitation after TKA by literature research and consulting experts from various disciplines.

**Methods:**

The Delphi method was used. Based on literature research and analysis, a set of candidate indicators was developed. Fifteen experts from different disciplines evaluated the validity of the indicators and provided modification suggestions.

**Results:**

After two rounds of Delphi consultations, consensus was reached. Agreement scores ranged from 4.40 to 5.00, with low variability (standard deviation 0.00–0.91) and high consistency (coefficient of variation 0.00–0.20). *P* was less than 0.05 in Kendall’s W with an agreement rate of 80.00–100%. In the final set of indicators, there were five primary indicators (representing four swelling causes and a general category), along with 23 secondary indicators and 40 tertiary indicators.

**Conclusions:**

This study preliminarily established indicators for at-home identification of post-TKA swelling caused by four distinct reasons. Further research is needed to validate the value of these indicators in distinguishing the causes of swelling.

## Background

Total knee arthroplasty (TKA) is commonly performed to alleviate pain and improve joint function in patients with end-stage knee osteoarthritis [1]. The number of TKA surgeries has been steadily on the rise due to its efficacy [2]. However, lower limb swelling is a common complication following TKA, with the rate being reportedly as high as 90.7% at 2–3 weeks after discharge [3]. Swelling negatively impacts postoperative rehabilitation, leading to slower restoration of muscle strength and delayed recovery of range of motion (ROM) [4, 5]. It also contributes to longer timed up and go (TUG) time at 6 weeks postoperatively [6]. Furthermore, the duration of postoperative swelling after TKA lasts longer than expected. Previous studies have shown that knee circumference remains 11% greater than preoperative levels even at 90 days postoperatively [5]. Wood et al. [7] reported a knee joint swelling rate of 40.0% at 6 months and 26.9% at 1 year after TKA, with swelling in the calf occurring at 23.9% at 6 months and 19.1% at 1 year. In a survey of residual symptoms in young TKA patients at 1–4 years postoperatively, 33% of the patients reported persistent swelling [8]. Therefore, swelling often occurs during the home-based rehabilitation phase after discharge, rather than solely during the hospital stay.

TKA patients are predominantly elderly people, and managing swelling during the home-based rehabilitation phase presents challenges for both patients and their families [9]. If swelling during home-based rehabilitation requires hospital visits, it poses significantly more burdens, physically, socially, and financially, on patients and healthcare resources. Thus, it is crucial/urgent to explore remote management solutions for addressing such swelling. One approach is to collect easily accessible data and patients’ self-reported symptoms, to allow for monitoring and targeted intervention of swelling. Previous research indicated that remote monitoring of TKA patients offered benefits by promoting postoperative recovery while reducing the need for frequent hospital visits, thus optimizing healthcare resource utilization [10]. The application of mobile health (mHealth) and computer-based technologies for TKA patient rehabilitation and remote monitoring have been reported, with most of such systems being used for tracking pain, activity, exercise adherence, range of motion, and gait analysis [11]. However, due to the lack of data regarding home-based swelling monitoring indicators, the study on this aspect has been scanty.

To improve the swelling symptom management module in mHealth systems, it is essential to develop effective intervention strategies based on swelling monitoring results. The major challenge is to differentiate the various causes of swelling. Our preliminary research has demonstrated that postoperative swelling after TKA could result from multiple factors, including inflammatory response, poor venous return, joint hematoma, and muscle damage and healing, among others [12]. Prior studies on swelling management have yielded inconsistent results or unfavorable outcomes [13–16], which might be attributed to the failure to identify the underlying cause of the swelling before intervention. As a result, the intervention failed to address the mechanism by which the swelling occurs. It is imperative to first identify the cause of the swelling before starting any intervention measures to maximize the efficacy of the intervention.

However, to our knowledge, no published studies identified indicators for monitoring and discriminating swelling after TKA during home-based rehabilitation. The absence of such indicators renders it challenging to develop precise swelling intervention plans and implement effective interventions. In this study, we aimed to bridge this gap by identifying indicators for monitoring and distinguishing between the causes of swelling after TKA during home-based rehabilitation through a literature review and the Delphi method. The findings of this study will be conducive to the development of personalized remote monitoring systems for home-based rehabilitation patients and improving postoperative outcomes.

## Methods

The Delphi method was adopted [17]. To ensure methodological rigor and transparency, the recommendations provided by the CREDES (Guidance on Conducting and Reporting Delphi Studies) were followed [18].

### Research team

The research team consisted of an orthopaedic nurse (team leader), an orthopedic director, a nursing management specialist, and two orthopedic nurses. The team analyzed the literature, designed the expert consultation questionnaire, and evaluated the results of the questionnaire study.

### Development of the first draft of the indicators

Several databases, including PubMed, Web of Science, CINAHL, China National Knowledge Internet (CNKI), Wanfang Data, and China Science and Technology Journal Database (Sinomed) were systematically searched to retrieve literature related to the external characteristics and influencing factors of the causes of different TKA postoperative swelling. The search terms were based on the purpose of the study and the results of our previous studies, including: (“knee replacement” OR “knee arthroplasty”) AND (“swell*” OR “edema” OR “oedema”) AND (“trial*” OR “factor*”). All publication years were covered, up to November 20, 2022. The retrieved literature underwent deduplication and screening. During the process of developing the first draft of the indicators, a comprehensive full-text review of the included literature was conducted, and studies that reported the following were considered for inclusion in the first draft: (1) specific manifestations or concomitant symptoms of each type of swelling. (2) factors underlying different types of swelling. (3) intervention strategies for swelling. The research team further discussed the measurability of these indicators in the home setting. Only indicators that were both relevant to identifying swelling and measurable at home were included in the first draft.

The draft was organized into three levels. Since the aim of the study was to develop indicators for identifying the causes of various types of swelling, the first-level indicators were mainly derived from the causes of swelling from previous studies. In addition, a “general category” was introduced as a first-level indicator to accommodate those indicators that did not fall into any particular category of swelling causes. The secondary or second-level indicators were the characteristics or influencing factors corresponding to the various categories of swelling obtained from the literature. Tertiary indicators were set as additional details to the secondary indicators when the secondary indicators needed to have a more granular categorization. For example, if the secondary indicator was “medication usage”, the tertiary indicator further specified the type of medication, e.g., antibiotics, hormones, NSAIDs, so that specialists could understand and advise more clearly.

### Development of expert consultation questionnaire

The questionnaire distributed to experts for consultation contained three parts as follows.

1. The first part described the questionnaire and covered the background of the study, the purpose and significance of the study, the process of constructing the first draft of the indicators, the purpose of the expert consultation, the components of the questionnaire, the expected return time of the questionnaire, and the contact information of the researcher.
2. The second part was the body of the questionnaire, i.e., the first draft of the indicators. A five-point Likert-type scale was applied in the study, ranging from strong agreement to strong disagreement. If there were any modification comments, such as suggestions for adding, deleting, or modifying the items, they could be made in the column of “*the modification comments*”.
3. The third part contained the basic information questionnaire of the experts, including age, gender, department, years of work, education level, academic title, whether they are postgraduate supervisors, and main research fields. Understanding this information was conducive to the assessment of the diversity of perspectives within the expert panel.

### Selection of experts for consultation

In line with the principles of the Delphi method and the research objectives, apart from orthopedic surgeons specializing in TKA, experts from other fields were also included. Rehabilitation medicine specialists and physiotherapists were included due to their extensive practical experience in managing post-TKA swelling. To gain further insights from a nursing perspective, nursing specialists from orthopedics were also included, as they had more direct contact with patients and closely monitored the symptoms. Vascular surgeons and nursing specialists were included because the causes of post-TKA swelling include impaired venous return, an area in which vascular surgeons have more specialized knowledge and experience. Internal medicine experts were also included, as some cases of lower limb swelling might not be directly caused by TKA but rather associated with the patient's underlying conditions. The selection criteria for the experts were: (i) extensive practical or research experience in the field related to lower limb swelling after TKA, with 10 years or more of work experience; (ii) bachelor's degree or higher; (iii) intermediate or higher technical title; (iv) having demonstrated enthusiasm for participating in this study and willingness to follow the principle of informed consent. Our research team conducted internal discussions and initially identified expert candidates based on predefined selection criteria, preferentially enrolling those who held positions in domestic and international professional associations. This approach was intended to ensure a broader perspective, making their opinions more representative. The main researchers then reached out to the identified experts, explaining the study objectives, the significance of their expertise in the field, and the expected time commitment, while inquiring about their willingness to participate in the study. Interested experts were required to confirm their willingness to participate.

### Implementation of expert consultation

**Fig. 1:**
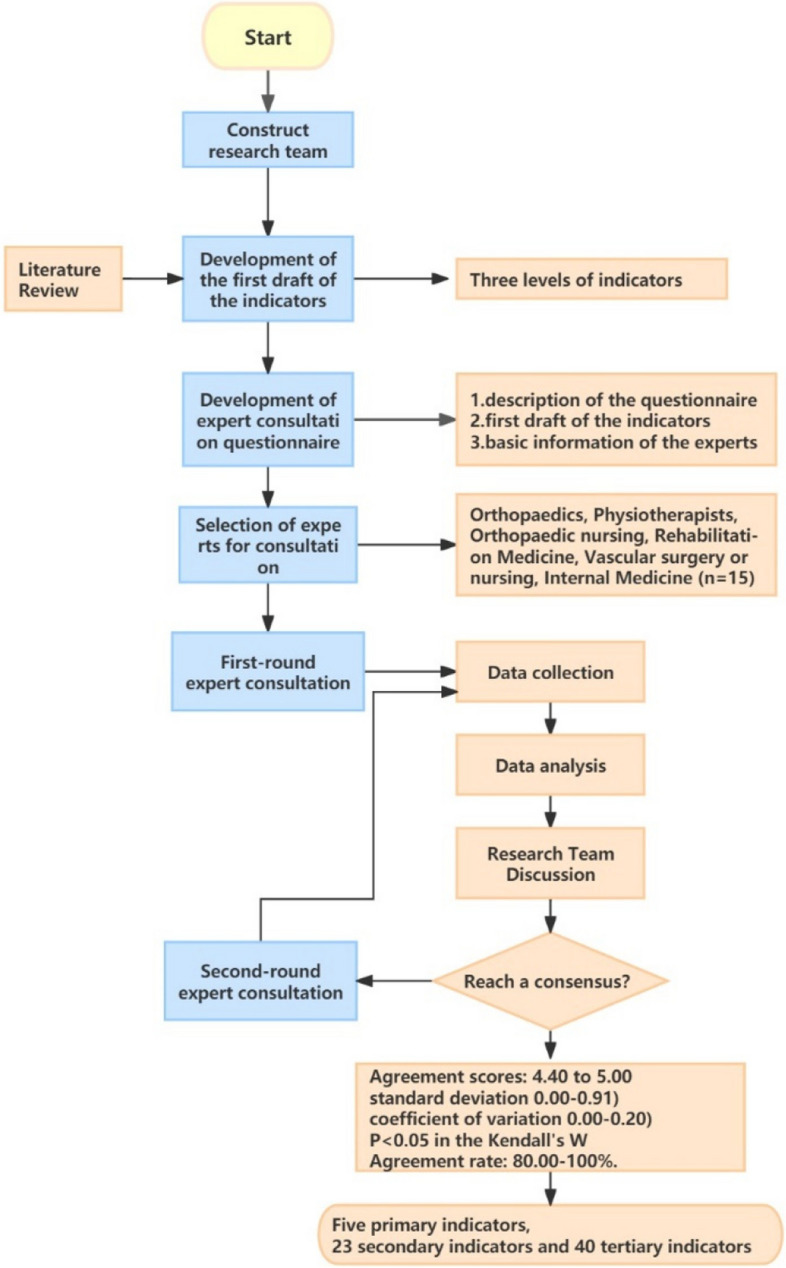
Flow diagram of the study

The principal investigators initially contacted experts via email or the WeChat app, distributing questionnaires and setting a two-week deadline for their completion. Follow-up communication was conducted by the researcher in cases where experts did not respond within the specified timeframe to inquire about progress and reasons for the lack of response. Upon receiving the completed questionnaires from the first round, the main researcher analyzed the experts’ opinions and facilitated a research team discussion. Indicators were evaluated based on criteria including SD < 3.5 and CV > 0.25. Indicators were added, removed, or modified based on the feedback provided by the experts. Using the results of the first round, the next round of expert consultation questionnaires was designed. The modifications made based on the first-round consultation were presented to the experts to ensure transparency. The same distribution and collection process was followed, and the results were analyzed accordingly. The consultation process continued until a consensus was reached among the experts, resulting in the completion of two rounds of expert consultation. The entire process is detailed in Fig. 1.

### Statistical analysis

The data were analyzed by using SPSS 22.0 software. The basic expert profile was presented in terms of mean, standard deviation (SD), frequency, and percentage. The mean and the percentage of agreement were applied to measure the convergence of the experts’ opinions. The SD, the coefficient of variation (CV), and Kendall’s coefficient of concordance (Kendall’s W) represented the divergence of the experts’ opinions. A mean score of 4, an 80% agreement, and a *P* < 0.05 in Kendall’s W were the criteria for consensus in this study [19].

## Results

A total of 15 experts were selected for consultation, with an age range of 35 to 59 years and an average age of (42.87 ± 7.20) years. The experts had a working experience ranging from 10 to 38 years, with an average of (19.13 ± 7.73) years. Table 1 shows the basic information of the experts.

**Table 1:** Basic information of experts (*n* = 15)

Items	Groups	*n*	%
Gender	Men	7	46.7
	Women	8	53.3
Ages	≤ 40 years old	8	53.3
	41–50 years old	5	33.3
	51–60 years old	2	13.3
Education	Bachelor’s degree	6	40.0
	Master’s degree	2	13.3
	PhD	7	46.7
Technical title	Intermediate	8	53.3
	Associate Senior	3	20.0
	Full Senior	4	26.7
Years of work	10–19 years	9	60.0
	20–29 years	4	26.7
	30 years and above	2	13.3
Research/work area	Orthopedics	3	20.0
	Physiotherapists	4	26.7
	Orthopedic nursing	4	26.7
	Rehabilitation Medicine	1	6.67
	Vascular surgery or nursing	2	13.3
	Internal Medicine	1	6.67
Graduate Student Mentor	Doctoral program Supervisor	3	20.0
	Master’s degree advisor	4	26.7

### Degree of expert motivation

The positive degree of experts was determined by the questionnaire return rate, which was 100%. During the first round, 6 experts provided 12 suggestions for revision, while in the second round, 2 experts offered 3 suggestions. These results indicated a strong interest in the study and a relatively high level of motivation among the experts. Two rounds were needed to reach a consensus. Agreement scores and ratings in each stage are illustrated in Table 2.

**Table 2:** Agreement scores and rate in each round

Value	1st Round	2nd Round
Agreement score^a^		
Mean	3.07–4.80	4.40–5.00
SD	0.56–1.71	0.00–0.91
Coefficient of variation	0.12–0.56	0.00–0.20
Kendall’s W**	X^2^ = 0.261 *P* < 0.001	X^2^ = 0.146 *P* < 0.001
Agreement rate (%)^b^	53.33–93.33	80.00–100

^a^Agreement score: “Strong agreement = 5, Agreement = 4, Neutral = 3, Disagreement = 2, Strong disagreement = 1” for each item

^b^Agreement rate: The percentage of the participants choosing “Strong agreement” and “Agreement” for each item

^**^P < 0.05 means “The agreement scores of all the items from all the participants have reached consensus

### Modification of the indicators

During the first round of expert consultation, eight (53.3%) experts gave 15 comments. Indicators were revised based on indicator screening criteria, literature analysis, and research team discussions.

1. Revision of 3 indicators: For the secondary indicator of “joint hematoma”, the term “bleeding tendency” was changed to “bleeding”, as experts suggested that the expression of bleeding tendency was not accurate and that only bleeding could be observed, such as subcutaneous bleeding, nasal mucosal bleeding, or gastrointestinal bleeding. For the tertiary indicator of “degree of swelling”, the experts in rehabilitation medicine suggested that the term “mid-patellar” should be changed to “knee joint”, and that the term “10 cm below the patella” should be revised to “the thickest part of the calf”, as these are technically or academically used expressions.
2. Addition of 8 indicators: Nursing experts suggested adding a tertiary indicator “mode of pain occurrence” to “pain in different parts of the lower limbs” to allow for a more comprehensive assessment of pain. Rehabilitation medicine experts suggested including “20 cm above the patella”, “15 cm above the patella” and “5 cm above the patella” as measurements of lower limb circumference to enable a more comprehensive evaluation of lower limb swelling. Internal medicine specialists recommend the addition of a history of underlying diseases, such as “kidney disease” and “liver disease” to differentiate them from surgery-related swelling, as these conditions can also cause lower limb swelling. Orthopedic surgeons and nursing specialists recommended the inclusion of medication reports for “non-steroidal anti-inflammatory drugs (NSAIDs)” and “anti-swelling medication”, as these are also commonly used in the postoperative period and can have an impact on the outcome of swelling.
3. Deletion of 5 indicators: Based on SD < 3.5 and CV > 0.25, the secondary indicator “emotion” and its tertiary indicator “anxiety self-assessment scale” under the first-level indicator “inflammatory response” were deleted, as experts did not reach a consensus on the impact of anxiety on inflammatory response mechanisms. The secondary indicator “other interventions” and its tertiary indicators “manual lymphatic drainage” and “kinesiology taping” under the indicator “poor venous return” were deleted, possibly due to the unclear effectiveness of these interventions and their limited utilization in clinical practice.

During the second round of expert consultation, 2 experts (13.3%) provided 3 suggestions for modification. Based on discussions within the research team, 2 indicators were removed. The secondary indicator “muscle activity” under the category of “muscle injury and healing” was eliminated because it was difficult for patients to measure. The tertiary indicator “temperature” under the category of “ice therapy” was also removed due to the limited feasibility of patients measuring and reporting this indicator. The final indicators for Monitoring and Discriminating Swelling during Home-based Rehabilitation after TKA, including five primary indicators, 23 secondary, and 40 tertiary indicators, are detailed in Table 3.

**Table 3:** Indicators for Monitoring and Discriminating Swelling during Home-based Rehabilitation after TKA

Primary indicators	Secondary and tertiary indicators
1. Inflammation response	
	1.1 Knee joint skin temperature
	1.2 Body mass index (BMI)
	1.3 History of underlying diseases
	1.3.1 Diabetes mellitus
	1.4 Medication usage
	1.4.1 Antibiotics
	1.4.2 Hormones
	1.4.3 NSAIDs
	1.5 Cold therapy
	1.5.1 Duration
2. Poor venous return	
	2.1 Pain in different parts of the lower limbs
	2.1.1 Pain score
	2.1.2 Onset of pain
	2.1.3 Mode of pain occurrence
	2.2 Deep venous thrombosis risk score
	2.3 Skin temperature
	2.3.1 Knee joint
	2.3.2 Foot dorsum
	2.4 History of underlying diseases
	2.4.1 Lower extremity venous disease
	2.4.2 Cardiovascular disease
	2.4.3 Disease-related limb paralysis
	2.4.4 Lymphatic system disease
	2.4.5 Hematologic disorders
	2.5 Medication usage
	2.5.1 Anticoagulant
	2.6 Compression therapy
	2.6.1 Method
	2.6.2 Duration per day
	2.7 Ankle pump
	2.7.1 Frequency
	2.8 Body position
	2.8.1 Duration in supine
	2.8.2 Duration in semi-recumbent
	2.8.3 Duration in sitting
	2.8.4 Duration in standing
	2.8.5 Duration of walking
3 Joint hematoma	
	3.1 Medication usage
	3.1.1 Anticoagulant
	3.1.2 Hemostatic
	3.2 Bleeding
	3.2.1 Location and area
	3.3 Wound drainage
	3.4 Tourniquet usage
	3.4.1 Pressure
	3.4.2 Duration
	3.5 Ice therapy
	3.5.1 Duration
	3.6 Knee ROM
4 Muscle injury and healing	
	4.1 Quadriceps muscle strength
5 General Category	
	5.1 Lower limb circumference
	5.1.1 20 cm above the patella
	5.1.2 15 cm above the patella
	5.1.3 10 cm above the patella
	5.1.4 5 cm above the patella
	5.1.5 Knee joint
	5.1.6 Thickest part of the calf
	5.1.7 Ankle
	5.2 History of underlying diseases
	5.2.1 Kidney disease
	5.2.2 Liver disease
	5.3 Medication usage
	5.3.1 Anti-swelling medication

## Discussion

The questionnaire achieved a high recovery rate of 100% in both rounds of consultation, indicating the experts’ strong motivation and engagement in the study. The experts were sourced from various fields including orthopaedics, rehabilitation medicine, physiotherapy, internal medicine, vascular surgery, and nursing. This diverse representation ensured a multidisciplinary approach to providing professional recommendations for constructing the indicators. After two rounds of Delphi consultations, consensus was reached. Agreement scores ranged from 4.40 to 5.00, with low variability (standard deviation 0.00–0.91) and high consistency (coefficient of variation 0.00–0.20). *P* < 0.05 in the Kendall's W with an agreement rate of 80.00–100%. In conclusion, the indicators constructed in this study demonstrated some scientific validity, and future research efforts will empirically verify which indicators are effective in identifying various types of swelling.

### Inflammatory response

Skin temperature of the knee has been included as an indicator of the external characteristics of the inflammatory response, as inflammation often leads to a localized increase in temperature.

Many researchers have explored localized elevated skin temperature after TKA, which can indicate the presence of an inflammatory response [20, 21]. Skin temperature is also easy to measure and obtain at home with the use of a handheld infrared thermometer, so this indicator was included.

Gao’s study [22] showed that BMI is an influential factor in postoperative limb swelling, which may be related to the fullness of subcutaneous adipose tissue, decreased skin elasticity, and adequate tissue fluid permeability interstitials in obese patients and that obesity also induces a low-grade systemic inflammatory state [23]. And BMI was also included as it can be calculated and obtained only by measuring the height and weight of the patient. Diabetes triggers excessive release of inflammatory cytokines and abnormal elevation of inflammatory mediators in the body, contributing to an increased inflammatory response after surgery. Therefore, the history of diabetes was included as an indicator, which can be obtained through patient self-report. The administration of antibiotics, hormones, and NSAIDs is common during intra- or postoperative periods to inhibit inflammatory responses and prevent infections. Patients may also use these medications due to their underlying disease, which can be reported by the patients themselves. Cold packs have been shown to inhibit the release of inflammatory cytokines, reduce the metabolic rate in the affected area, and mitigate the inflammatory response [24, 25]. Patients can provide information on the duration of cold pack applications during their home rehabilitation process.

### Poor venous return

According to the opinion of vascular surgery specialists, when lower limb swelling is the result of poor venous return, it may signal the development of deep vein thrombosis (DVT), which can be accompanied by sudden onset of pain, severe swelling of the entire lower extremity unilaterally, and elevated skin temperature. Therefore, we included pain scores, time of onset, and mode of attack of different lower limb sites (thigh, calf, and knee), and skin temperature of the knee and foot dorsum as indicators. Pain scores can be assessed using a numeric rating scale (NRS) that allows patients to rate pain on a scale of 0–10 points [26].

Several underlying conditions are also associated with the development of venous reflux insufficiency and so were included as indicators to be reported by the patient. Preoperative venous insufficiency in patients affects postoperative swelling, and ultrasound assessment of the saphenous vein diameter (GSV) > 5.38 mm and saphenous venous reflux duration > 1.23 s had predictive value for the development of postoperative lower limb swelling after TKA [27]. Cardiovascular disease affects circulation and fluid balance in the lower limb, disorders of the lymphatic system may cause lymphatic fluid to accumulate in the tissues, hematologic disorders may cause a state of hypercoagulability of the blood, and paralysis due to other disorders increase the risk of poor venous return, so a history of all these underlying disorders is included in the indicators.

The level of risk of postoperative DVT in patients with TKA can be obtained from the score of the Caprini risk assessment model, and the level of the score can be used to predict the risk of swelling, which is also recommended by the guidelines [28, 29], and routinely assessed by the clinic, and this metric is also included. For patients at high risk of DVT, the guidelines recommend the need for physical and pharmacologic anticoagulation to reduce the risk of DVT [29], but if bleeding occurs with medication, the risk may need to be assessed and consideration is given to discontinuing the medication, so anticoagulant use will need to be documented.

Previous interventional studies have shown that the use of compression (elastic bandage or stocking) and ankle pumping exercises after TKA can promote venous return to the lower extremities, but the effectiveness of compression bandage use in reducing swelling has not been demonstrated [13, 30]. Another study of a new compression regimen (combined use of compression stockings and self-adjustable non-elastic bandages), worn continuously for 6 weeks, showed that although it had no effect on swelling in the postoperative 0–2 days, but reduced swelling in the postoperative 14 days [31], suggesting that different modes of compression may have an effect on outcome. Therefore, the patient's compression mode and duration, and the number of activities of ankle pump movement were included as indicators.

When patients stand, walk, or sit for prolonged periods, the combined impact of gravity, impaired venous valve function, and reduced muscle pump function can lead to inadequate venous return in the lower limb, thereby increasing the risk of swelling. So, indicators capturing the duration of time spent in different positions were incorporated to collect data and investigate their influence on swelling.

In the first draft, we obtained some other interventions from the literature to reduce swelling, such as unarmed lymphatic drainage and kinesiology taping [32, 33], but were deleted after expert consultation due to the high heterogeneity of the scores, probably due to the uncertainty of the effectiveness and limited use in clinical practice. However, there may be geographical differences in the frequency of use in practice, and further high-quality studies could be conducted in the future.

### Joint hematoma

The occurrence and extent of joint hematomas can be influenced by the use of medications, particularly anticoagulants and hemostatic drugs commonly employed in therapy, so these indicators were included. Wound drainage was included as an indicator, considering its potential to reduce the incidence of early hematomas. However, it is worth noting that studies on other fracture disorders have shown the possibility of hematoma re-formation after drain removal [34]. So, it was deemed necessary to explore whether wound drainage could similarly impact the occurrence of joint hematomas following TKA.

The duration and pressure of tourniquet use were included as indicators that may impact joint hematoma formation. While tourniquet usage helps reduce intraoperative blood loss, provides improved surgical visualization, and shortens the surgical duration, it also triggers local ischemia, hypoxia, local inflammatory reactions, and reperfusion injuries, which are important mechanisms contributing to postoperative limb swelling after TKA. A systematic evaluation demonstrated that the difference in knee circumference between the intraoperative tourniquet group and the no tourniquet group was significantly greater at postoperative days 1, 3, 5–21, and weeks 3–6 (*P* < 0.05), with no significant difference observed at postoperative months 4–6 [35]. The impact of tourniquet duration and pressure on outcomes has also been explored [36–39], but no definitive recommendations have been established. Therefore, these indicators were included to investigate the relationship between tourniquet use and swelling outcomes.

Ice therapy can induce local vasoconstriction, reduce vasospasm and blood flow, and decrease bleeding [24, 25], so the use of ice therapy was also included in the indicators related to joint hematomas. The inclusion of knee ROM as an indicator is based on the understanding that increased ROM may exert additional compression on the traumatized tissues, thereby elevating the risk of vascular injury and bleeding, potentially leading to the formation of joint hematomas.

### Muscle injury and healing

Quadriceps muscle strength is included as the next level indicator of muscle injury and healing. Muscle injuries during TKA can mainly result from tourniquet use and surgical incisions [40]. The medial parapatellar approach (MPA) is the classic surgical approach for TKA, which is less difficult to perform and has good exposure to the surgical field, but longitudinal dissection of the quadriceps tendon causes injury [41]. The assessment of quadriceps muscle strength can be conducted using unarmed muscle strength assessment techniques, employing the Lovett grading method for evaluation.

### General category

Circumference measurements at different sites of the lower limb were mandatory to be included, as represent a fundamental aspect of swelling monitoring. In response to the comments from the rehabilitation medicine experts, the measurement site indicators provided in the draft were supplemented and modified, and seven indicators for lower limb sites were finally included (20 cm above the patella, 15 cm above the patella, 10 cm above the patella, 5 cm above the patella, knee, thickest part of the calf, and ankle). These indicators contribute to a more comprehensive understanding of postoperative lower limb swelling after TKA. Although it is possible that some of these indicators may have limited impact after data analysis, given the limited existing evidence on postoperative swelling changes after TKA, we opted to collect these data comprehensively to inform future studies. In our previous review of tools for assessing swelling, we found that tape measurement has the lowest cost, whereas bioelectrical impedance analysis may yield more precise results, necessitating the selection of the appropriate tool based on the availability of resources [42].

Internal medicine specialists recommend including kidney and liver disease history as indicators to differentiate between swelling caused by these diseases or related to TKA. Other medications that affect swelling were included in the indicator, such as sodium aescinate, which is recommended by orthopaedic surgeons and commonly used in clinical practice. Its pharmacological effects involve a combination of anti-inflammatory properties and improved venous blood flow, making it suitable for inclusion in the general category of indicators rather than being specific to a particular cause of swelling.

### Limitations

It is important to acknowledge the limitations of the indicator system developed in this study. First, the initial indicators were derived from a literature review, with a thorough search of common databases. However, expanding the search to include more databases could yield different results. Future research could broaden the search scope to obtain additional evidence. Additionally, all experts consulted were from the same hospital in China, which may limit the generalizability of the indicators due to regional differences in knowledge and medical practice. To mitigate this, the selected experts hold positions in various international and domestic professional associations, ensuring their perspectives are broader and scientifically valid. Given the multidisciplinary nature of the topic, experts from different fields were included. Our sample size met the methodological requirements of the Delphi approach; however, it is undeniable that including a larger number of experts could potentially lead to more transferable results. Stakeholders such as patients and caregivers were not included in the study. Although their firsthand experiences with swelling are valuable, their limited understanding of disease-related expertise could result in less meaningful contributions. Nonetheless, their perspectives might offer potential value, particularly in improving the feasibility of indicator collection. In future research phases, particularly during the development of remote monitoring tools, the input of patients and caregivers could be incorporated to enhance the practical application of the indicators. These limitations should be considered when applying the indicators in practice.

### Implications for practice

The indicators identified in this study offer valuable insights for clinical practice and may contribute to the future development of remote monitoring for swelling after TKA. While some of these indicators are already collected by clinical practice or exist mHealth systems [11], they are seldom used to monitor swelling. There was a lack of models to support the development of swelling monitoring modules. Our findings provide a reference for constructing models to identify and predict the causes of swelling. Based on the indicators included in the model and considering data collection feasibility and patient preferences [43], wearable devices could be further developed and integrated into mHealth systems. Existing mHealth systems have been reported to alert care teams when a decrease in physical activity or slower-than-expected progress is detected, enabling timely intervention. This feature allows caregivers to adjust rehabilitation plans or carry out necessary medical procedures, ensuring an efficient and personalized recovery while avoiding potential complications or delays [43]. This functionality could also be applied to manage swelling symptoms. For patients who are prompted to seek medical attention, clinicians could also utilize longitudinal swelling data to track recovery progress and assist in diagnosing complications. With the advancement of mHealth technologies and big data, these indicator data will offer valuable insights for future research.

## Conclusion

This study employed a draft based on evidence obtained from a literature review and a Delphi method with multidisciplinary expert consultation to develop indicators for monitoring and differentiating swelling during home rehabilitation after TKA. Our findings suggest that collecting data on swelling characteristics, influencing factors, and interventions can help identify the causes of postoperative swelling, leading to more targeted intervention recommendations. Further research is needed to develop models for identifying and predicting the causes of swelling while simplifying the indicators to enhance data collection feasibility and cost-effectiveness. To augment the swelling management module in mHealth systems, tools like wearable devices could be developed for remote data collection and symptom monitoring.

## Data Availability

The datasets used and analysed during the current study are available from the corresponding author on reasonable request.
